# Simultaneous Pancreas and Kidney Transplantation in Patients With Type 2 Diabetes Mellitus

**DOI:** 10.3389/ti.2025.14772

**Published:** 2025-10-07

**Authors:** Ruth Owen, Jack Reynard, Emily Thompson, Georgios Kourounis, Chris Snowden, Angeles Maillo-Nieto, James Shaw, Colin Wilson, Steve White

**Affiliations:** Institute of Transplantation, Freeman Hospital, Newcastle Upon Tyne, United Kingdom

**Keywords:** T2DM, equity, outcome predictors, SPK transplantation, review of literature

## Abstract

The prevalence of diabetes is increasing exponentially, accompanied by an increase in chronic complications, including nephropathy. Kidney transplantation may offer freedom from dialysis but adding a pancreas addresses the underlying disease. Type 2 diabetes mellitus (T2DM) is often described as a condition of insulin resistance and the concurrent beta-cell loss and dysfunction is potentially underestimated. The aim of this review was to provide a critical appraisal of simultaneous pancreas and kidney (SPK) transplantation in recipients with T2DM. The primary concern with SPK transplantation in this group is insulin resistance and the impact of obesity on outcomes. Multiple studies have shown comparable graft survival (GS), patient survival and complication rates when comparing T2DM and T1DM recipients. Furthermore, patients with T2DM had significantly improved GS with SPK when compared to kidney transplantation alone. Despite these findings, SPK transplantation is only selectively used in T2DM patients. Existing literature focuses on comparing transplant outcomes between patients with T1DM and T2DM. We believe the more relevant question is whether a patient with T2DM would derive a meaningful benefit from an SPK, and whether these benefits outweigh the risks, in the context of their other co-morbidities which are not completely similar to those associated with T1DM.

## Introduction

Diabetes mellitus represents a significant health challenge with approximately 525million individuals affected worldwide as of 2021 [1]. It is a major cause of blindness, end-stage renal disease (ESRD), cardiovascular events, cerebrovascular accidents, limb amputation and premature mortality. It exerts a substantial economic strain on healthcare systems and accounts for approximately 9% of the annual health budget spent in Europe (€149billion) [2, 3].

Persistent hyperglycaemia leads to renal failure through inflammation, increased vascular permeability and hypertrophy of podocytes [4]. Diabetes-associated renal disease is thought to occur in 30%–50% of T2DM patients and 15%–30% of those with T1DM [5–8]. Patients with T2DM tend to present with concurrent renal disease as they have often had diabetes for years prior to diagnosis. There is, unsurprisingly, an association with progression to ESRD in patients with T2DM and concurrent co-morbidities (age and hypertension) [9]. For T2DM patients with ESRD, treatment includes dialysis, renal transplantation or, in rare cases, SPK transplantation.

The first SPK transplantation was performed in 1966 by Kelly and Lillihei [10]. Their objective was to restore kidney function and to provide an endogenous source of insulin, enhancing glucose control, eliminating the need for insulin injections, and preventing further end-organ damage secondary to hyperglycaemia. At the time, it seemed pointless to treat one condition and not the other, although this was considered very controversial with their contemporaries. Subsequent research has shown that a pancreas transplantation (PTx) can improve native kidney function, potentially reversing the pathological changes of diabetic nephropathy [11], providing further justification for a simultaneous pancreas and kidney transplantation approach.

The first SPK transplantation recipient had T1DM and, since inception, has rarely been offered to T2DM patients. It was assumed that a PTx would be less beneficial in these recipients, as the recipient’s insulin resistance would prevent the new source of insulin from being effectively utilised. As a result, the treatment focus for T2DM has been on medical therapies aimed at improving insulin sensitivity, secretion and promoting glycosuria, such as thiazolidinediones, glucagon-like peptide-1 (GLP-1) receptor agonists, and sodium, glucose cotransporter-2 inhibitors (SGLT2i). These medications are increasingly being used in combination with a kidney transplant alone and have been suggested to reduce all-cause mortality and potentially HbA1c [12, 13]. There is a perception, which is reasonable, that drug treatments carry far less risk than a major surgical operation such as a PTx, even if the patient has ESRD and needs a kidney transplant, but it is often overlooked that adding a pancreas in the right patient would be more effective in the long-term.

This review aims to consolidate current literature regarding SPK transplantation outcomes in patients with T2DM, compare outcomes after transplant with alternative therapies and comment on current listing criteria.

### Type 2 Diabetes

In the 1930s, clinicians identified two primary forms of diabetes: one characterised by immediate insulin dependence, typically in younger individuals, and another occurring later in life, often with obesity, where insulin therapy was not initially required for survival [14]. This distinction led to the Type 1 and Type 2 terms we are familiar with today.

Early research in T2DM largely focused on the mechanisms underlying insulin resistance, often overlooking, or in some cases completely ignoring, the role of beta cell loss, dysfunction and insulin deficiency. A study using cadaveric pancreas tissue showed obese T2DM patients had up to 60% loss of beta cell mass compared to obese patients without a diabetes diagnosis [15]. It is well appreciated for those involved in beta-cell therapy that hyperglycaemia causes glucose toxicity in the pancreas causing oxidative stress and beta cell dysfunction [16]. This results in a multiplier effect where the pancreas is less able to produce insulin, glucose levels increase and further exacerbate the glucose toxicity effects and beta-cell dysfunction.

It is clear, the initial understanding of T2DM was too simplistic and it is not a homogenous disease but a polymorphic condition with various genetic, metabolic and clinical characteristics. A deeper understanding of this heterogeneity suggests the initial binary categories do not adequately reflect the disease. Newer subtypes have been described including severe-insulin-deficient diabetes (SIDD) and severe-insulin-resistant diabetes (SIRD) [17]. This classification may represent a way to identify T2DM patients with lower insulin resistance for which there is a greater potential benefit for PTx.

In the UK, diabetes is primarily diagnosed in primary or emergency care based on clinical presentation and fasting blood glucose levels. Diabetes-related autoantibodies and C-peptide measurements are not standard practice. Management pathways diverge dependent on classification. National Health Service (NHS) guidance recommends urgent referral to an endocrinologist for those with T1DM, while T2DM patients are typically managed in primary care with National Institute for Health and Care Excellence (NICE) guidance outlining a stepwise treatment approach, beginning with lifestyle modifications, then oral agents, and eventually insulin therapy if glycaemic control remains inadequate. Many T2DM patients never undergo specialist evaluation, risking misclassification, particularly in the cohort of patients with concomitant beta-cell exhaustion who may quickly progress to insulin treatment and simply be misdiagnosed as having poorly controlled T2DM, especially if they are overweight [18–20].

A key concern with PTx in T2DM is the degree of insulin resistance, and whether the pancreas graft would be able to overcome this. It was previously thought that a PTx in a T2DM patient would be subject to overstimulation and resultant islet exhaustion and allograft failure. However, multiple studies have shown that insulin secretion and sensitivity are improved in these patients [21–24]. The gold standard technique for measuring insulin resistance is the glucose clamp technique [25]. This is a labour intensive, invasive test and simply not practical in a routine clinical setting. Predictive models may be able to quantify insulin resistance in a more accessible manner. One is the homeostatic model assessment of B-cell function and insulin resistance (HOMA-IR) [26]. A patient’s fasting glucose level is compared with the models’ predictions to estimate insulin resistance. The newer subtypes SIDD and SIRD use HOMA for classification.To the authors knowledge there have been no published studies looking at HOMA in the pre-operative period as a predictor of outcomes, relating to T2DM and SPK transplants. Other predictive models include QUICKI [27] and METS-IR [28]. Limitations of using predictive models include their generic nature and lack of individual results. They also often poorly represent certain populations [29]. HOMA-IR should be used with caution in patients with low BMI [30] and women >50 years old [31]. Further research into the value of these models in the pre-transplant assessment is needed.

C-peptide has limited value for patients being considered for SPK. It is a connecting peptide cleaved during the production of insulin and has been used as a surrogate marker of insulin production [32]. It is also renally excreted and filtered during dialysis [33] complicating interpretation within the patient population who would be eligible for SPK transplant. A patient who is C peptide positive always generates debate when considering beta cell replacement therapy, but its presence is not an absolute contra-indication if the patient is insulin dependent [22]. A 2023 study by the Wisconsin group analysed the impact of pre-transplant C-peptide on SPK transplant outcomes in T2DM [34]. Patients were delineated into low (<2 ng/mL), medium (2–8 ng/mL) and high (>8 ng/mL) C-peptide levels. The group reported excellent outcomes across all groups with comparable uncensored and death-censored kidney graft failure. Adjusted C-peptide levels increased in all groups following pancreas transplant. This group advised against making clinical decisions to exclude patients from SPK transplant based on C-peptide levels, in particular high C-peptide levels.

When critically appraising the literature, it is clear the method of classification of T2DM is not standardised. Some rely on C-peptide levels [23, 35] and titres of diabetes auto antibodies, others use primarily a clinical diagnosis [36, 37]. There has even been a novel scoring system created [38]. Taken together this makes useful conclusions often difficult to judge.

### Selection Criteria for SPK in Type 2 Diabetes

In the UK, eligibility for SPK transplantation follows the NHS Blood and Transplant (NHSBT) patient selection policy POL185/6 [39]. Criteria include insulin dependence and dialysis or an eGFR below 20 mL/min at the time of listing. For T2DM patients, a Body Mass Index (BMI) ≤30 kg/m^2^ is required and a higher BMI considered an absolute contraindication to transplant. Current guidelines do not mandate testing for insulin resistance, glucose tolerance tests, or C-peptide levels. As an alternative, patients with T2DM are not usually eligible for an islet transplant in the UK unless they are insulin dependent and have a BMI <30, the same indications for a pancreas transplant.

The BMI limitation for T2DM patients is a point of contention. Obesity has been linked to post-operative complications such as transplant pancreatitis, graft thrombosis, and poorer wound healing [40, 41]. However, our group found a BMI >30 kg/m^2^ was not associated with increased risk of complications [42]. This study also showed that whilst recipient BMI was an independent risk factor affecting graft and patient survival after PTx, the exact value at which this should become a barrier to transplant was not definable even in a very large cohort of patients. BMI is a poor surrogate for body composition and may inadequately reflect appropriateness for transplant. Alternative measurements such as body girth or hip-waist ratio, may be more relevant but again needs to be defined [43, 44].

Furthermore, patients with BMI >30 kg/m^2^ who have pathological weight-loss secondary to ESRD may eventually meet BMI criteria [45] but having years of dialysis beforehand will make them more frail and less fit for transplantation, we know that pre-emptive transplantation have the best outcomes after SPK. Spain also uses a BMI <30 kg/m^2^ [46] and the cut-off in the US is slightly higher at 35 kg/m^2^ [47]. In the authors opinion the latter sounds more reasonable because other transplants have higher relative cut offs e.g. liver transplantation [48] is often set at 40 kg/m^2^ [49, 50] as is renal transplantation.

### Outcomes of SPK Transplant in T2DM

Our group have also previously reported graft and patient survival after SPK delineated by type of diabetes (n = 2,060) [36]. 3.4% (n = 94) of transplants were performed in T2DM recipients. Diabetes was pre-defined by the listing centre using clinical criteria which has scope for bias reporting in such a heterogenous group. NICE guidance uses a fasting plasma glucose >7 mmol/L and clinical features such as ketosis, rapid weight loss and autoimmune history to diagnose and distinguish T1DM from T2DM [51]. This study showed comparable patient survival at 1 year (T1DM:96.8%, T2DM:96.5%) and 3 years (T1DM:93.2%, T2DM:89.3%) regardless of diabetes type. At 5 years we saw a statistically significant decrease in T2DM patient survival (T1DM:89.4%, T2DM:79.2%), but this trend was not borne out at 10 years (T1DM:74.8%, T2DM:73.1%). Pancreas and kidney graft survival was comparable at all time points and there was no difference in complications including cardiac events and post-operative infections.

Other studies have been identified from electronic databases including Ovid MEDLINE database, PubMed and google scholar using the terms “simultaneous pancreas and kidney transplant,” “T2DM,” “SPK,” detailed in Table 1.

**TABLE 1:** Studies pertaining to T2DM and SPK Transplant.

Study Year and Author	Type of Study	No of transplants and breakdown of type	Salient Findings
2024 Parajuli [88]	Single-centre Wisconsin, US cohort	SPK transplant only N = 183 4 groups A^+^β^–^, A^–^β^–^, A^–^β^+^, and A^+^β^+^	T2DM Diagnosis Criteria Patients were stratified by autoantibody status and pre-transplant fasting C peptide A^+^ detection of ≥1 autoantibody A^–^ no autoantibodies detected β^–^ fasting C-peptide <2 ng/mL β^+^ fasting C-peptide ≥2 ng/mL Those A^–^β^+^ would represent patients with T2DM Results Pancreas and kidney graft survival was comparable irrespective of stratification
2024 Martinez [89]	Single-centre Wisconsin, US cohort	SPK transplant only N = 345 13.6% T2DM	T2DM Diagnosis Criteria T2DM – older age at diabetes diagnosis, prior use of oral glycaemic agents, absence of auto antibodies, detectable C-peptide T1DM – younger age of diagnosis, presence of autoantibodies, absence of C-peptide Results Comparable patient, kidney-graft and pancreas-graft survival was noted when comparing patients with T1DM and T2dM Comparable rates of readmission post-transplant, comparable rates of SSI, comparable rates of major surgical complications and thrombosis
2024 Owen [36]	Multicentre UK cohort	SPK transplant only N = 2,236 3.4% T2DM	T2DM Diagnosis Criteria Type of diabetes was defined by clinical diagnosis T2DM – older age of onset, metabolic features, initial use of oral glycaemic agents T1DM – ketosis, younger age of onset, lower BMI, immediate insulin use Results Comparable graft survival at 1 year, 3 years, 5 years and 10 years Comparable patient survival at 1 year, 3 years and 10 years Statistically inferior patient survival at 5 years - trend not borne out at 10 years, nor in multivariable model No difference in complication incidence between groups
2023 Parajuli [34]	Single-centre Wisconsin, US cohort	SPK transplant only N = 76 Delineated by pre-transplant C-peptide level Low n = 14 Medium n = 47 High n = 15	T2DM Diagnosis Criteria Novel scoring system giving score from −9 to +9, a negative score correlated with T2DM and a positive score with T1DM. Results Excellent outcomes after SPK transplant for all recipients Comparable rates of uncensored and death-censored kidney graft failure irrespective of pretransplant C-peptide level Post-transplant C-peptide levels increased in all groups after SPK when adjusting for the patients renal function
2022 Amara [90]	Systematic Review	Pancreas and islet transplant Studies publishing original data from 2000 onwards	T2DM Diagnosis Criteria This review utilised studies defining T2DM with any recognised criteria including C-peptide, BMI, absence of ketoacidosis, absence of autoantibodies and age at diagnosis Results 5 studies compared patients with T2DM undergoing SPK to those with T2DM undergoing KTA. SPK was suggested to have superior outcomes in these studies 17 studies compared patients with T2DM undergoing SPK to those with T1DM undergoing SPK and found comparable outcomes (93.75% of studies)
2021 Pham [38]	Single-centre Wisconsin, US cohort	SPK transplant only N = 323 12.1% T2DM	T2DM Diagnosis Criteria Novel scoring system giving score from −9 to +9, a negative score correlated with T2DM and a positive score with T1DM. Results Comparable pancreas and patient survival No association found between BMI and post-transplant diabetes mellitus No association found between pre-transplant insulin requirements and post-transplant diabetes mellitus
2020 Hau [91]	Single-centre Lepzig, German cohort	SPK and KTA N = 127 70.1% T1DM SPK 9.4% T2DM SPK 20.5% T2DM KTA	T2DM Diagnosis Criteria: Diagnosis with either · Diagnosis age >40, no history of ketoacidosis and either a weight >115% ideal body weight or non-consistent insulin therapy for 2 years after diagnosis · Diagnosis age 30–39, no history of ketoacidosis, weight >115% ideal body weight and non-consistent insulin therapy for 2 years after diagnosis Results T1DM and T2DM recipients who received an SPK transplant there were comparable graft and patient survival Recipients with T2DM who received a KTA had poorer graft and patient survival when compared to SPK but it should be noted had statistically significant differences in demographics (were older and more comorbid) limiting comparison
2020 Alhamad [92]	Multicentre US cohort	SPK and KTA N = 35,849 100% T2DM 2% SPK	T2DM Diagnosis Criteria Pre-defined by the OPTN database, no further details are provided Results Statistically significantly superior kidney graft and patient survival for patients with T2DM who received an SPK when compared to KTA (deceased or living donor)
2019 Rohan [93]	Single-centre South Carolina, USA cohort	SPK, PTA, PAK SPK n = 91 41.8% T2DM	T2DM Diagnosis Criteria Detectable C-peptide level Results Note - outcomes are for all types of pancreas transplant and there was no specific SPK subgroup analysis Comparable glycaemic control post-transplant between T1DM and T2DM recipients Comparable complication rates (including infections, rejection, graft loss and patient survival) Those with T2DM had a higher incidence of BK virus nepropathy
2019 Liu [94]	Single-centre, Guangzhou, China cohort	SPK transplant only N = 63 29% T2DM	T2DM Diagnosis Criteria Not defined Results Comparable rates of complications (delayed kidney graft function, kidney rejection, pancreatitis, pancreas rejection, duodenal leak, pancreatic fistula, portal thrombosis, intestinal obstruction) Comparable pancreas graft, kidney graft and patient survival was noted
2019 Andacoglu [95]	Single-centre, Washington US cohort	SPK, PTA, PAK SPK n = 34 25% T2DM SPK	T2DM Diagnosis Criteria T2DM – detectable C-peptide, age at diagnosis, and BMI Results Comparable glycaemic control between T1DM and T2DM at 2 years post-transplant
2019 Shin [96]	Single-centre, Seoul, Korean cohort	SPK, SPLK, PAK, PTA SPK and SPLK n = 99 22% T2DM	T2DM Diagnosis Criteria: Diagnosed by either · Diabetes onset after age 40 and either weight >115% of ideal body weight or no consistent insulin use in the first 2 years after diabetes diagnosis · Diabetes onset between 30 and 40 years old and both a weight >115% of ideal body weight and no consistent insulin use in the first 2 years after diabetes diagnosis Results Comparable metabolic outcomes in patients with T1DM and T2DM after pancreas transplant (all forms and subgroup analysis of SPK only), this included HbA1c levels, C peptide levels on HOMA-IR scores
2018 Gondolesi [97]	Single-centre Buenos Aires, Argentina cohort	SPK and PTA n = 46 PTA n = 1 24.5% T2DM (All SPK)	T2DM Diagnosis Criteria T2DM - >30years/o at time of diagnosis with metabolic features T1DM - diagnosis in childhood with a high ketone levels and immediate insulin treatment Results No statistically significant difference in patient survival at 1 year or 5 years
2017 Gruessner [98]	Multi-centre, International cohort	SPK. PTA, PAK N = 1,514 100% T2DM (n = 1,317 SPK)	T2DM Diagnosis Criteria Definition provided that “the recommendations of the American Diabetes Association were used to check and correct classification of diabetes type” Results SPK transplant is a safe procedure in patients with T2DM with a 95% survival at 1 year
2016 Chakkera [21]	Single-centre prospective observational study, Minnesota, USA	SPK transplant only N = 16 43.8% T2DM	T2DM Diagnosis Criteria T1DM – Undetectable C-peptide (<0.1 ng/mL) on insulin therapy from the time of diagnosis. T2DM (referred to as non-T1DM in the text had a detectable C-peptide and a history or oral agents with progression to insulin Results Similar metabolic profile (determined using HOMA-IR score) between T1DM and select T2DM patients Comparable measures of glucose homeostasis at 1 year between T1DM and T2DM
2016 Jeon [99]	Two-centre, Seoul, Korean cohort	LD KTA, DD KTA, SPK, Dialysis SPK n = 48 49.6% T2DM SPK	T2DM Diagnosis Criteria T1DM defined as undetectable C-peptide (<0.8 ng/mL) and the presence of anti-pancreatic or anti-insulin autoantibodies, T2DM defined as patients not defined by the criteria above Results Patient survival was superior in patients receiving any form of transplant than dialysis alone Patient survival was statistically significantly better in those undergoing LD KTA, compared with DD KTA and SPK. It was highlighted that the waiting time for SPK in Korea is very long which may explain these results Comparable patient, kidney graft and pancreas graft survival when comparing T1DM and T2DM receiving an SPK
2013 Light [23]	Single-centre Washington, US cohort	SPK transplant only N = 173 33.5% T2DM	T2DM Diagnosis Criteria T1DM defined as undetectable C-peptide (<0.8 ng/mL), T2DM defined as detectable C-peptide (>0.8 ng/mL) Results Comparable time until first rejection in those patients that experience rejection Statistically significant poorer patient survival in recipients with a detectable c-peptide
2013 Margreiter [35]	Single-centre Innsbruck, Austria cohort	SPK and KTA N = 248 78.6% T1DM SPK 12.9% T2DM SPK 8.5% KTA	T2DM Diagnosis Criteria T1DM diagnoses as early onset, immediate insulin requirement, presence of autoantibodies and C-peptide negativity. T2DM diagnosed with C-peptide level Results No statistically significant difference in pancreas graft survival Poorer patient survival in recipients with T2DM receiving an SPKT compared with T1DM recipients (univariate), not borne out in multivariable model This dataset also compared T2DM receiving KTA compared with T1DM & T2DM receiving SPK. Those receiving KTA had inferior 5-year graft (p < 0.001) and patient (p < 0.0001) survival
2012 Park [100]	Single-centre Seoul, Korean cohort	SPK, PTA, PAK SPK n = 91 17.5% T2DM	T2DM Diagnosis Criteria Not defined in the text Results No statistically significant difference in pancreas graft survival
2011 Sampaio [37]	Multicentre US cohort, OPTN/UNOS data	SPK transplant only N = 6,756 8.6% T2DM	T2DM Diagnosis Criteria Serum C-peptide >0.8 ng/mL Results Delayed kidney graft function and primary non function rates were statistically higher in T2DM recipients Pancreas complication rates were comparable Death censored kidney graft survival was poorer in patients with T2DM (p = 0.04) Comparable patient survival Comparable pancreas graft survival
2010 Chakkera [21]	Single centre Minnesota, US cohort	SPK transplant only N = 80 12.5% T2DM	T2DM Diagnosis Criteria Defined patients as T2DM if they had detectable C-peptide, negative GAD65 Antibody, absence of diabetic ketoacidosis and the use of oral hypoglycaemics Results Comparable graft and patient survival between recipients with T1DM and T2DM
2005 Nath [101]	Single centre Minnesota, US retrospective observational analysis	SPK, PTA and PAK N = 17 7 (41%) SPK 100% T2DM, no comparison with T1DM	T2DM Diagnosis Criteria: Diagnosed by either · Diabetes onset after age 40 and either weight >115% of ideal body weight or no consistent insulin use in the first 2 years after diabetes diagnosis · Diabetes onset between 30 and 40 years old and both a weight >115% of ideal body weight and no consistent insulin use in the first 2 years after diabetes diagnosis Results 1 year patient and graft survival rate was 94%, and all surviving patients were euglycemic at 1 year

KTA: Kidney Transplant Alone, LD: Living Donor, PAK: Pancreas After Kidney, PTA: Pancreas Transplant Alone, SLPK: Simultaneous deceased donor pancreas and Living donor Kidney transplant, SPK: Simultaneous Pancreas and Kidney transplant, T1DM: Type 1 Diabetes Mellitus, T2DM: Type 2 Diabetes Mellitus, UNOS: United Network for Organ Sharing.

The largest (n = 6,756), utilised the United Network for Organ Sharing (UNOS) database [37]. Most patients who received an SPK (90.8%, n = 6,141) had T1DM and much fewer, (8.2%, n = 582) had T2DM. This study also showed comparable death-censored graft survival at 5 years (T1DM:85.3%, T2DM:83.0%) and patient survival was said to be comparable but exact figures were not provided. The type of diabetes in this study was again predefined by the listing centre. The American Diabetes Association (ADA) use a fasting random plasma glucose >11.1 mmol/L and a Hba1C >48 mmol/mol for diagnosis. Characteristics such as age, BMI, presence of other autoimmune diseases and history of ketoacidosis aided distinguishment between T1DM and T2DM.

There are also multiple single centre studies comparing SPK recipient outcomes delineated by type of diabetes. The Wisconsin group, (n = 323) defined T2DM using clinical judgement [38]. They demonstrated comparable pancreas graft survival (death-censored) and incidence of post-transplantation diabetes mellitus (PTDM) between recipients with T2DM (n = 39) compared with T1DM (n = 284). The patients were well matched with comparable BMI, age and sex. A novel scoring system was used to confirm diabetes type and looked at; pre-transplant insulin requirement, pre-transplant fasting c-peptide levels (assigning a score of +2 if C-peptide <0.5 ng/L, −1 if 0.5–2 ng/L and −2 if >2 ng/L), family history and the presence of diabetes-associated antibodies. A score from −9 to +9 was created for each recipient, and a negative score defined as T2DM and a positive score with T1DM. Again, this showed comparable patient or graft survival. It would also be interesting to better understand the reclassification rate—i.e., how many patients had their diabetes type changed after applying the novel scoring system. This information was not provided but would be especially relevant given the joint consensus statement by the ADA and the European Association for the Study of Diabetes (EASD), noting that up to 40% of those diagnosed with T1DM after age 30 were initially misclassified as T2DM [52].

A smaller single centre study was reported in 2013 by an Austrian group (n = 248) comparing T1DM undergoing SPK transplant (n = 195) with T2DM SPK (n = 21) and also with T2DM receiving a kidney transplant alone (KTA) (n = 32) [35]. They defined T2DM using detectable C-peptide levels. They also ensured a minimum of 6 months oral therapy prior to being started on insulin in their diagnosis and had a BMI cut off >32 kg/m^2^. Comparable rates of pancreas graft survival between T1DM and T2DM recipients undergoing SPK were described. A statistically significant poorer patient survival (PS) was seen in univariate analysis when comparing T2DM recipients who had an SPK with T2DM recipients who had a KTA and with T1DM recipients who had an SPK at 1 year (T2-SPK:90.5% T2-KTA:87.1%, T1-SPK:96.9%) and 5 years (T2-SPK:80.1% T2-KTA:54.2%, T1-SPK:91.6%). A multivariable analysis was performed adjusting for donor and recipient age, BMI, Cold Ischaemic Time (CIT) and patient survival was no longer statistically significantly different. This univariate finding contrasts with the other literature discussed. It is also important to note this paper does not differentiate KTA by donor brain death (DBD), donor circulatory death (DCD) or living related donor (LRD) which can makes accurate analysis difficult.

From 2004 to 2019, only 3.4% of SPK transplants in the UK were performed in patients with T2DM [36]. Other countries have comparable proportions of patients with T2DM; 91% in USA [53], 90%–95% in Germany [54] and 90% in the Netherlands [55]. In 2010 the International Pancreas Transplant Registry, IPTR (which receives data from both UNOS and Eurotransplant) showed 8% of SPK’s were performed in T2DM patients [56]. The 2024 Annual report of OPTN/SRTR showed almost a quarter of SPK transplants were performed in patients with T2DM [57], suggesting the UK is more stringent in accepting patients with T2DM for SPK. However, a positive trend in the UK is noted with an increase in the percentage of total SPK’s being performed in patients with T2DM each year (2% 2005, 4.3% 2009, 5.8% 2018) [36]. Reasons explaining the relatively lower numbers are not entirely clear.

Graft and patient survival are not the only metrics of success, and it is imperative to look further at post-transplant glycaemic control after transplant, renal function, and quality of life. A Chinese study assessed renal function and HbA1C after KTA and SPK in recipients with T2DM, using propensity score matching [58]. This study found that 2 years post-transplant, those who had an SPK transplant had a statistically significantly greater decrease in HbA1C (HR:1.05, 95%CI: 0.7–10.4, p = 0.005), decreased fasting blood glucose (HR:2.49 95%CI: 1.81–3.17, p < 0.001), decreased triglyceride levels (HR:0.65, 95%CI: 0.39–0.91, p = 0.0015) and a higher eGFR (HR:-14.5, 95%CI −18.6–−10.4, p < 0.001) than those who had a KTA.

There are no studies looking specifically at quality-of-life or patient reported outcomes (PROMS) after SPK in T2DM, and these should be a focus for further research. An American study (n = 54) compared T1DM recipients who underwent KTA compared to SPK [59]. They found improved diabetes-related quality of life (QOL) scores (using the Diabetes QOL questionnaire [60]) in SPK recipients and equivalent mental health and wellbeing scores utilising the Medical Outcome Health Survey Short Form-36.

### Comparison of SPK With Alternative Therapies for Patients With T2DM

When considering SPK transplant, alternatives should always be evaluated, including remaining on dialysis, kidney transplant alone with wearable technologies.

#### Dialysis

Dialysis provides a method of filtration and excretion. However, dialysis has significant morbidity long-term including peritoneal infections and fistula complications. For those on haemodialysis, it often prevents patients being a part of the workforce and being economically inactive is associated with low self-esteem and poor mental health [61, 62].

#### Kidney Transplant Alone

A KTA has a well-established survival and quality of life advantage over dialysis and is an option for patients with T2DM and end stage renal failure [63]. However, this will not address the primary issue of hyperglycaemia and as such nephropathy may later occur in the donated kidney [64, 65]. It should be noted that as medical management of hyperglycaemia improves, the benefit of the addition of a pancreas, may reduce.

A Taiwanese group recently published a propensity matched study assessing the use of SGLT2i inhibitors after kidney transplantation in diabetic recipients [12]. This landmark study showed a significant improvement in all-cause mortality, (2.08% in the SGLT2i user compared to 9.54% in the non-user group at 3.4 years - their median follow-up time), a reduction in major adverse cardiac events (SGLT2i: 4.44% compared to non-SGLT2i: 13.87%, HR 0.48) and a reduction in major adverse kidney events (SGLT2i: 8.93% compared with non-SGLT2i: 22.54%, HR 0.52). They noted that only 6.5% of kidney transplant recipients with diabetes utilise a SGLT2i and so there is significant scope for implementation.

A US trial (the FLOW trial) evaluated the impact of Semaglutide use in patients with T2DM and an eGFR between 25 and 75 mL/min/1.73 m^2^ [66]. The use of this GLP-1 receptor agonist was seen to slow the decline of renal function, as reduced the risk of cardiovascular events and death. It should be noted that the effect of Semaglutide on these outcomes was thought not to be relate to changes in body weight, but to a potential of decreasing inflammation, oxidative stress and fibrosis in the kidney. A further study was performed assessing the use of GLP-1 agonists in kidney transplant recipients and also found improved graft and patient survival with the use of a GLP-1 agonist [67].

The other consideration is the additional risk that presents with the addition of a pancreas transplant. Recipients undergoing SPK have more episodes of rejection than those with a KTA and so are treated with more aggressive immunosuppression regimes [68]. These patients also have higher rates of wound infections and urinary tract infections [69]. Pancreas grafts can have enteric leaks, bleeding, and small bowel obstruction, all leading to higher morbidity and mortality in these patients [70].

#### Wearable Insulin Technologies

Wearable insulin sensors and pumps have revolutionized diabetes management, enabling personalised insulin regimes and reducing needlestick burden [71, 72]. NICE guidance was changed in 2023 to allow adults with T2DM to be offered continuous glucose monitoring, prior to this they were excluded [73]. We hope these technologies will have a significant impact on diabetic complications in future but there is limited data demonstrating benefit in transplant patients. This is one area that needs urgent attention. Given the challenges that immunosuppression brings to managing blood glucose [74, 75], it is imperative that these technologies are prioritised for transplant patients.

### Challenges for SPK Transplant for Patients With T2DM

#### BMI and Obesity as Barriers to SPK in T2DM

Pre-transplant weight loss strategies include diet, exercise, bariatric surgery and GLP1-inhibitors.

Diet and exercise regimens may be challenging for patients with renal failure due to electrolyte imbalances associated with fruit, vegetable and protein intake [76]. Exercise regimens may also be difficult secondary to fatigue often present in chronic illness. A US study followed 376 patients who were BMI >30 kg/m^2^ and asked to lose weight prior to being listed for transplant. Only 10% of patients lost any weight at all and a meagre 5% reached their target weight [44]. This challenges the efficacy of these programmes and raises ethical concerns about delaying listing for transplantation when success is limited.

Bariatric and metabolic surgery (BMS) may be an option [77]. A recent meta-analysis found that BMS (gastric bypass, sleeve gastrectomy, gastric banding and duodenal switch) was both safe and efficacious, with a combined mortality rate for patients who underwent both BMS and KTA of 4% which is not much different in non-obese patients having KTA or even SPK transplantation in expert centres [78]. They recommended that it formed part of the transplantation work-up process to enable hard-to-list obese patients to be considered. A smaller Minnesotan study followed 17 patients who underwent bariatric surgery prior to PTx (11 gastric bypass, 5 sleeve gastrectomy and one gastroplasty). Post-operatively the median BMI decreased from 37.4 kg/m^2^ to 26.4 kg/m^2^ and the median time from bariatric surgery to transplant was 2.4 years. These patients were compared with control matched patients and had comparable length of stay, graft thrombosis and incidence of rejection. At the 4-year follow up time, graft and patient survival was 100%, suggesting that in the right patients it should be considered [79]. It is important to note that whilst bariatric surgery may facilitate transplant it also has the potential to negate the need for SPK transplant. Multiple studies have shown that BMS improves glucose haemostasis and could lead to diabetes remission [80, 81].

Obese patients with T2DM may benefit from the use of GLP-1/GIP analogues such as Semaglutide or Tirzepatide to lose weight [82, 83]. These analogues enhance insulin secretion, inhibits glucagon release, slows gastric emptying, and promotes satiety, which leads to weight loss and improved insulin sensitivity. Weight loss may also decrease anaesthetic risks, particularly around intubation and cardiovascular events. In the post-operative period weight reduction may improve wound healing. However, it is important to note that GLP-1/GIP analogues should be used with caution and careful monitoring, as it can have gastrointestinal side effects such as nausea (53.3%) and vomiting (30.3%), which could lead to dehydration [83]. There are case reports suggesting Semaglutide may cause pancreatitis which could be catastrophic in the context of a transplanted pancreas graft [84, 85]. However, a recent meta-analysis showed no increased risk [86]. The other concern is the predisposition for muscle loss [87]. In the renal failure population, already at risk of sarcopenia, GLP-1/GIP analogues use would need to be delivered with considerable dietician oversight.

#### Post-Transplant Complications

Renal failure and suboptimal glucose control are well-documented risk factors for myocardial infarction (MI) and impaired wound healing. These risks, however, are not unique to T2DM patients and are similarly observed in individuals with T1DM. Our analysis, consistent with prior studies, revealed no significant difference in the incidence of postoperative complications between T2DM and T1DM recipients undergoing SPK transplant [36].

## Conclusion

SPK transplantation is a complex procedure requiring careful patient selection to ensure benefits outweigh risks. It offers freedom from dialysis, insulin independence and improved quality of life. At present, patients with T2DM show improved HbA1c after transplant and superior kidney graft survival when compared to a KTA. Improvements in medical management of hyperglycaemia may reduce the benefit of the additional pancreas when compared to KTA and should be re-evaluated regularly. QOL outcomes remain unexplored in this cohort. Predictive models (such as HOMA-IR) may identify T2DM patients with low insulin resistance who could benefit from SPK transplant.

Most literature focuses on comparing outcomes with patients with T1DM. We hypothesise that there is much greater overlap in the pathophysiology of T1DM and T2DM and many complications and comorbidities are similar. We believe the more appropriate questions should be, is there a recipient with T2DM that would benefit from a SPK transplant? Are the risks acceptable? Then, if the benefits outweigh the risks, listing is justified.

There is a desire for clearer guidelines as to which recipients with T2DM should receive an SPK if more patients were to be accepted for transplant. The data is limited by the smaller number of SPK transplants performed in T2DM patients, and most studies are underpowered to provide statistical confidence. Our group would support the cautious expansion of SPK transplant to patients with T2DM using the current listing criteria as outcomes after transplant remain satisfactory.

## Abbreviations

ADA, American Diabetes Association; BMI, Body Mass Index; BMS, Bariatric and Metabolic Surgery; CIT, Cold Ischaemic Time; DBD, Donation after Brain Death; DCD, Donation after Circulatory Death; ESRD, End stage renal disease; GLP-1, Glucagon-like peptide −1; GS, Graft Survival; HOMA-IR, Homeostatic Model Assessment for Insulin Resistance; KTA, Kidney Transplant Alone; LRD, Living Related Donor; MI -Myocardial Infarction; NHS, National Health Service; PROMS, Patient Reported Outcome Measures; PTDM, Post-transplant Diabetes Mellitus; PTx, Pancreas Transplant; SGLT2i, Sodium-Glucose Cotransporter 2 Inhibitors; SIDD, Severe Insulin-Deficient Diabetes; SIRD, Severe Insulin-Resistant Diabetes; SPK, Simultaneous Pancreas and Kidney Transplant; T1DM, Type 1 Diabetes Mellitus; T2DM, Type 2 Diabetes Mellitus; UNOS, United Network for Organ Sharing; QOL, Quality of Life.
